# Mechanism of the antidiabetic action of *Nigella sativa* and Thymoquinone: a review

**DOI:** 10.3389/fnut.2023.1126272

**Published:** 2023-09-25

**Authors:** Arslan Shaukat, Arsalan Zaidi, Haseeb Anwar, Nadeem Kizilbash

**Affiliations:** ^1^Department of Physiology, Government College University - GCU, Faisalabad, Punjab, Pakistan; ^2^National Probiotic Laboratory, National Institute for Biotechnology and Genetic Engineering College - NIBGE-C, Faisalabad, Punjab, Pakistan; ^3^Pakistan Institute of Engineering and Applied Sciences - PIEAS, Nilore, Islamabad, Pakistan; ^4^Department Medical Laboratory Technology, Faculty of Applied Medical Sciences, Northern Border University, Arar, Saudi Arabia

**Keywords:** diabetes mellitus, *Nigella sativa*, antioxidant, thymoquinone, anti-glycemic, gut microbiota

## Abstract

**Introduction:**

Long used in traditional medicine, *Nigella sativa* (NS; Ranunculaceae) has shown significant efficacy as an adjuvant therapy for diabetes mellitus (DM) management by improving glucose tolerance, decreasing hepatic gluconeogenesis, normalizing blood sugar and lipid imbalance, and stimulating insulin secretion from pancreatic cells. In this review, the pharmacological and pharmacokinetic properties of NS as a herbal diabetes medication are examined in depth, demonstrating how it counteracts oxidative stress and the onset and progression of DM.

**Methods:**

This literature review drew on databases such as Google Scholar and PubMed and various gray literature sources using search terms like the etiology of diabetes, conventional versus herbal therapy, subclinical pharmacology, pharmacokinetics, physiology, behavior, and clinical outcomes.

**Results:**

The efficiency and safety of NS in diabetes, notably its thymoquinone (TQ) rich volatile oil, have drawn great attention from researchers in recent years; the specific therapeutic dose has eluded determination so far. TQ has anti-diabetic, anti-inflammatory, antioxidant, and immunomodulatory properties but has not proved druggable. DM’s intimate link with oxidative stress, makes NS therapy relevant since it is a potent antioxidant that energizes the cell’s endogenous arsenal of antioxidant enzymes. NS attenuates insulin resistance, enhances insulin signaling, suppresses cyclooxygenase-2, upregulates insulin-like growth factor-1, and prevents endothelial dysfunction in DM.

**Conclusion:**

The interaction of NS with mainstream drugs, gut microbiota, and probiotics opens new possibilities for innovative therapies. Despite its strong potential to treat DM, NS and TQ must be examined in more inclusive clinical studies targeting underrepresented patient populations.

## The rising prevalence of diabetes mellitus and the need for new treatment approaches

DM is an ancient scourge, with evidence of its description first appearing in pre-biblical medical writings (1). It’s complicated pathophysiology consists of metabolic derangements marked by chronically elevated blood glucose, resulting from abnormalities in insulin action, secretion, or both (2–4). The name itself was coined in the 2^nd^ century CE derived from the Greek word “diabaínein” which means “a passing through,” alluding to the disease’s generally profuse urination. The term “mellitus” was added much later in the 16^th^ century because of the sugar found in the urine of people with diabetes (5, 6). In addition to its effect on sugar regulation, DM negatively impacts many important physiological processes, including fat and protein metabolism (2). Chronic hyperglycemia characteristic of DM correlates with long-term damage, dysfunction, and failure of multiple organs, including the eyes, kidneys, nerves, heart, and blood vessels.

The categorization of DM has long baffled medical professionals. Numerous classifications have been proposed, including the now-defunct insulin-dependent (ID) and non-insulin-dependent (NID) types of DM, which have been replaced with a nomenclature more consistent with the disease’s accepted etiology (7). DM is currently characterized clinically into four primary categories and two subtypes (8). Type 2 diabetes mellitus (T2DM) is the most prevalent worldwide, with estimates ranging from 85 to 95%, followed by type 1 (9, 10). The gestational and other diabetes types are rarer and entail a broader range of causes, such as pancreatitis, genetic defects, and endocrinopathies (2). Type 1 DM (T1DM) is distinguishable from T2DM because the former is an autoimmune disorder where pancreatic beta-cells are destroyed, while the latter is characterized by progressively dysfunctional glucose regulation attributable to a combination of insulin resistance and pancreatic beta-cell destruction (11, 12). T2DM and prediabetes are often associated with a broader disorder known as “metabolic syndrome” (13). Differentiating between these various diabetes types is challenging since more than one type can manifest in a single patient (13), prompting calls for a revision of how the disease is classified (2).

Diabetes mellitus (DM) morbidity and mortality have become serious worldwide health concerns in both developed and developing nations (14, 15) straining the world economy (16). Recent controversial claims argue that the disease burden has shifted to developing countries, exacerbating the problem (17, 18) because developing countries already house 79 percent of the world’s diabetes population (19), and the majority comprises of young people belonging to the lowest socioeconomic strata (20). Public health organizations tasked with epidemiological monitoring of DM have struggled to explain prediabetes’s soaring frequency and incidence and the seeming failure to diagnose the condition globally (21). Diabetes is a prime example of the so-called “over-nutrition disease” that is often connected with a surfeit of nutrients and dietary richness (22, 23). “Extra-nutritional” variables, such as the usage of bisphenol A (BPA) in food processing and packaging, also contribute to the spread of diabetes and accompanying comorbidities (24). Societies that have recently transitioned to modern lifestyles have been particularly hard hit by T2DM (14). Asians appear more vulnerable to T2DM, including those in Pakistan, where its incidence has exceeded projections (25). The entire subcontinent of South Asia has been labeled the “diabetes capital of the world” (26). Current estimates indicate the number of DM sufferers globally at 451 million; this figure is projected to rise to 693 million by 2045 (27). Additionally, a whopping 374 million are estimated to be prediabetic, a physiological state that usually leads to full-blown T2DM, a number that is projected to climb by an additional 200 million in the next decades (28). Due to their inadequate healthcare infrastructure, developing nations such as Pakistan will be the hardest hit by these dismal projections (25); thus, traditional medicine will assume even greater importance for the general population in the coming years (14, 29).

The deteriorating situation has spurred several recommendations, including from the WHO (30–32), to investigate the use of plant-based therapies for DM in conjunction with conventional treatments to sustainably and affordably combat the DM epidemic (14, 30, 33). The inability of Western medicine to produce a treatment for DM is another major factor driving the demand for novel alternative medication. Allopathy relies on managing DM with oral hypoglycemic and hypolipidemic drugs with suboptimal therapeutic outcomes and potentially severe side effects (15, 34). The four most common oral hypoglycemics, sulfonylureas/insulinotropics, biguanides, α-glucosidase inhibitors, and thiazolidinediones, have demonstrated efficacy but also safety concerns (35). Natural plant-derived compounds are being increasingly investigated as alternatives to synthetic antioxidants to safely treat numerous oxidative stress-related diseases and conditions (36). Due to their greater molecular variety compared to synthetics (37), medicinal plants have given many therapeutic compounds for treating human ailments, including antidiabetics (38–40).

## Methodology

A comprehensive literature search was conducted with publicly available web-based search engines and databases, PubMed, Scopus, ScienceDirect, Web of Science, Google Scholar, and other sources (R&D reports, graduate theses, and dissertations). The search was based on keyword combinations such as DM and etiology, oxidative stress and DM, herbal medicine and DM, challenges of conventional diabetes treatment, herbal medicine and DM, *N. sativa* and black seed oil, *N. sativa* and DM, *N. sativa* and phytoconstituents, thymoquinone and mechanism of action, antioxidant activity, gut microbiota, and DM. Research and review articles published in English from 1985 to 2023 were included. We also checked the references cited in the retrieved articles and reviews to avoid missing pertinent studies. The research articles were managed using EndNote software, version X9 (Thomson Reuters). Conference proceedings or abstracts, non-original research such as letters, protocols, editorials, commentaries, duplicated literature, clinical trials lacking robust controls, and papers dealing with homeopathic agents were excluded. A total of 481 relevant articles were found, which were exhaustively examined. An attempt was made to include literature from the disparate disciplines of human physiology, plant taxonomy, microbiology, and pharmacochemistry related to NS and its therapeutic potential in DM. The review incorporates the findings of 16 clinical trials.

## The role of herbal medicine formulations in DM treatment

The rebirth of interest in traditional herbal therapy is due, in part, to the progressively dwindling returns of the reductionist paradigm of drug development prevalent in the industry (25, 41, 42). A notable example is the ineffectiveness of conventional medications in treating chronic diabetes and their inability to address insulin sensitivity and secretion at the same time (43). Even metformin (metf), the US Food and Drug Administration’s (FDA) recommended front-line medicine for DM (44), is devoid of contraindications (45, 46), yet it fails to exert its therapeutic effect due to patient noncompliance (47). Herbal products, as part of the broader notion of integrative medicine, can be used to supplement standard allopathy or to completely replace it, a concept known as complementary and alternative medicine (CAM) (48). CAM is more commonly used in patients with chronic DM, with most patients preferring to supplement rather than replace their orthodox drug regimens (49). The holistic approach inherent in herbal medication gradually strengthens the body’s healing abilities and can better be described as preventive rather than curative.

Herbal therapy is based on the utilization of multi-ingredient formulations to achieve a combinatorial impact, with surprising effectiveness when compared to modern pharmaceutics, which is overwhelmingly centered on using single target molecules for treating complicated chronic condition like diabetes (50–52). According to WHO, 80 percent of the world’s population still relies on traditional medicine for healthcare (53), making it a legitimate element of the global healthcare network (54). Interestingly, many of the drugs used in modern medicine have botanical origins. The primary antidiabetic drug Metf was originally obtained from the French lilac *Galega officinalis* (44, 55). Certain plant materials that are rich in antioxidants, have been found effective in treating diabetes (56–58). A considerable body of evidence underscores the importance of herbal medicines in the treatment of diabetes (59), particularly those classified as spices (60–63). However, due to a lack of compelling evidence, many in the medical community remain skeptical of the utility of herbal medicine for DM treatment (64). This has prompted efforts to better understand the safety and efficacy of CAM products, practices, and interactions, often using radical trans-disciplinary approaches such as reverse pharmacology (49, 65).

It is worth noting that simply consuming antioxidant rich plants foods cannot be considered herbal therapy because the antioxidant molecules are enmeshed in the food matrix, where dosage and bioavailability can be problematic. Individual plant-derived antioxidants such as vitamins and polyphenols for diabetes treatment, on the other hand, have only partially succeeded due to stability issues and differences in the physiologies of lab animals compared to humans. Despite these challenges, it has been demonstrated that combining different antioxidants can have a synergistic effect, and such formulations are becoming increasingly popular (66). Because of the complexities of diabetes, where oxidative stress is so deeply intertwined with multiple metabolic pathways, therapies including herbal ones that have the twin capacity of antioxidant renewal and ROS route blocking would have the best chance of success (67, 68). Many front-line contemporary drugs currently used for diabetes treatment, such as thiazolidinediones, metf, and glucagon-like peptide-1 (GLP-1) agonists, owe their effectiveness to antioxidant activity and glucose-lowering capability (66).

### Taxonomy, biogeography, and ethnomedicinal importance of *Nigella sativa*

NS is an erect, annual flowering herb 20–90 cm tall, is one of the 20 species belonging to the genus *Nigella* L. (Family Ranunculaceae, Order Ranunculales, Class Magnoliopsida, Division Tracheophyta, Kingdom Plantae), accorded the taxonomic serial number of 506,592 by Integrated Taxonomic Information System (ITIS) (69), all the species withing this genus having utility as either food or medicine (70). The black tint of its discoid seeds occurs once they are exposed to air and is the source of many of its colloquial names “Black cumin” (71), “Black caraway” (72) “Alkamoun Alaswad” (73), or Black seed (74). Pertinent is the distinction between black cumin and black caraway from true cumin and true caraway, the latter two being the seeds of *Cuminum cyminium* L., and *Carum carvi* L, respectively, belonging to family Umbellifera (72). Despite the taxonomic impasse of dividing the Nigellae tribe into genera or sections, the consensus splits it into three genera, of which only *Nigella* is found in South Asia. *Komaroffia* and *Garidella*, on the other hand, are common throughout southern Europe and central Asia (74). NS stands out from the other *Nigella* spp. because its seed’s volatile oils are particularly rich in TQ (75), making it a promising candidate for both traditional, modern evidence-based phytomedicine with preventive and therapeutic potential (76–79). It remains one of the most widely researched medicinal herbs (80) and the one most frequently cited throughout medical history as the ultimate “cure-all” (81), having been described by the Greeks, the Bible (82), and in traditional Arab and Islamic medicine (TAIM) where it is exalted as a prophetic medicine (83). Besides having culinary value (84), its seeds and oil are believed to have holistic medicinal properties and are used in many ancient medicinal schemas such as Ayurvedic, Siddha, Unani, Chinese, and Islamic (76, 85–86). NS and its oil have been labeled GRAS (generally regarded as safe) by the USFDA for use as a spice (87) but given only a qualified GRAS approval for use as a dietary supplement (88). It is one of the most frequently purchased herbal supplements in many of the world’s leading markets (89). The Aegean and Irano-Turanian regions have been considered the evolutionary fountainhead of NS (90); however, a broader nativity claim is also made that includes North Africa and southwest Asia. Egypt produces the best commercial quality though it is also found growing wild in the Middle East, sub-continent, and Mediterranean countries (90, 91). India is its largest global producer (92). NS has an extensive array of pharmacological activities against various ailments and is considered a sacred herb in various religions, and a native, health-promoting plant in traditional medicine (78). In recent decades, researchers have found that NS has anti-inflammatory, and hepato-, neuro-, and gastro-protective effects (76, 78). It has also been substantiated that NS and its chemical constituents exert a nephroprotective effect by normalizing kidney function and reversing tubular damage along with suppression of biochemical alterations (93, 94).

### Chemical composition of NS

NS seeds (NSS) have been the focus of study since the latter part of the 19^th^ century (73) as they are the principal source of the herb’s bioactive components, and their volatile oil consists mainly of alkaloids, terpenes, and phenolics (90, 95). However, volatile oils comprise only 0.4–2.5% of the total seed content (96), the fixed oil being the principal part at 36–38% (87). The most active constituent is TQ (97, 98), discovered in 1963 (75). TQ makes up 18 to 57% of the volatile oil (96, 99), with the exact composition depending on species, seed chemotype, and oil extraction method (90, 100). The TQ content in NS oil (NSO) can be as low as 0.05 mg/ml to as high as 7.2 mg/ml (101). Some traditional processing practices employ solvents to effectively remove the TQ from NSS in an attempt to make them safer for people with specific health concerns (102). Other volatiles of interest are thymohydroquinone, pinene, p-cymene, and dithymoquinone. In addition to TQ, other potent radical scavengers include dithymoquinone, trans-anethole, thymol, and carvacrol, except dithymoquinone (90). In addition to these, carvacrol (5–12%), 4-terpinol (2–6%), and thymol are also present (83). NSS also contains limonene, citronellol, and two types of alkaloids, isoquinoline (nigellimin and nigellimin N-oxide) and pyrazole (nigelliden and nigellicin) (78, 87). TQ is present in several plant families besides the Ranunculaceae, but NS stands out as its richest source (103, 104), a claim worth investigating since members of the convergently co-evolved family Lamiaceae have shown amounts of TQ in their floral parts far exceeding those of NS (104, 105).

The essential oil of NSS is also high in polyunsaturated fatty acids, linoleic acid (50–55%), eicosanoic acid (4%), and monounsaturated fatty acids such as oleic (20%). NSS contains substantial phenolic compounds such as salicylic acid, quercetin, tocopherols, and phytosterols such as β-sitosterol (90).

### The nexus of DM and oxidative stress revisited

Oxidative stress (OS) is a central concept in biology and medicine. Since its introduction in 1985, it has evolved from a simple imbalance in oxidative species in cells and tissues (106) to a much more complex interaction between reactive oxygen species (ROS) and receptors, signaling pathways, and antioxidant defenses. It incorporates a loss of homeostasis in a cell’s many redox-driven physiological processes, defined as “an imbalance between oxidants and antioxidants in favor of the oxidants, leading to a disruption of redox signaling and molecular damage” (107). Depending on the degree of oxidative stress, it can be either harmful (distress) or helpful (eustress) (108). The pivotal role of redox control in cellular functions describing redox homeostasis as “aurea mediocritas” the golden mean of healthy life (109) has led many researchers to view T2DM as a redox disease (110).

OS is brought about by two broad classes of molecules, reactive oxygen species (ROS), such as H_2_O_2_, ·OH, and O2-and reactive nitrogen species (RNS), such as NO and NO_2_. These are naturally generated as byproducts of metabolic activities (111), but their accumulation can damage biological macromolecules like proteins, lipids, and nucleic acids (112). Under normal circumstances, with a robust antioxidant system, these molecules do not constitute a threat; only when the antioxidant response is compromised or oxidant levels rise too rapidly do they cause oxidative damage leading to diseases such as T2DM (113, 115). Free radicals are species containing one or more unpaired electrons, and this incomplete electron shell accounts for their high reactivity (116, 117). The most common free radical is superoxide anion (O_2_^−^.) (118), generated by the action of nicotinamide adenine dinucleotide phosphate (NADPH) oxidase (119). The most unstable and destructive is the hydroxyl radical (·OH) formed by the reaction of H_2_O_2_ with metal ions (Fenton reaction) which damages lipids through peroxidation, triggering a chain of adverse events (113).

**Figure 1:**
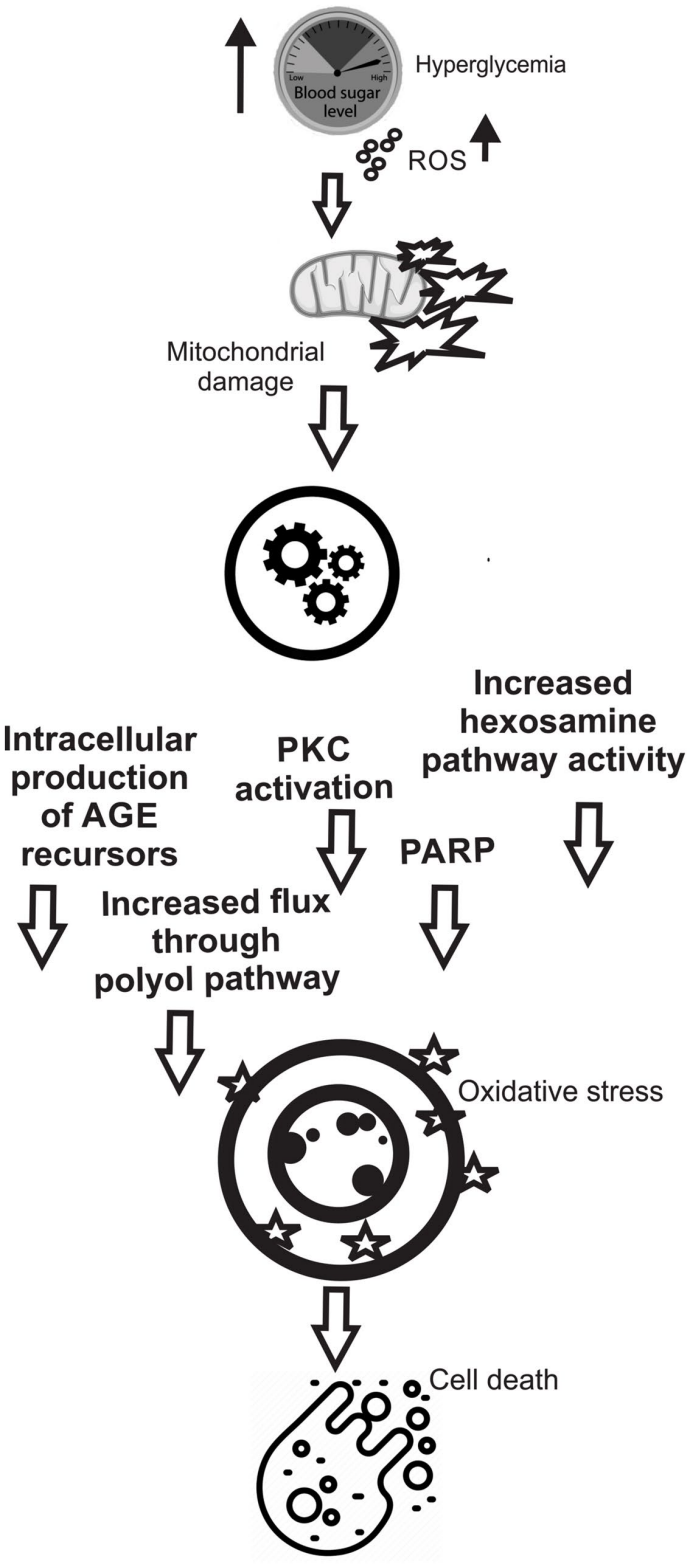
Various cellular pathways involved in hypoglycemia mediated ROS generation, mitochondrial damage, and cell death.

Peroxynitrite is a potent pro-oxidant (114) implicated as the causative agent of the cardiovascular endothelial dysfunction seen in T2DM (120). ROS can be generated via many pathways within cells; however, metabolic activities occurring in the mitochondria and endoplasmic reticulum and enzymes such as nicotinamide adenine dinucleotide phosphate (NADPH) oxidase are the usual causes associated with OS-mediated onset of T2DM (66) (Figure 1). However, it has been argued that terms like total antioxidant capacity (TAC) and reactive oxygen species (ROS) are too general and prone to misinterpretation when applied to the complex system of redox and oxidative components inherent in OS. Careful identification of the individual oxidant and antioxidant moieties and their behaviors allows for a better understanding of the mechanics of OS (121, 122).

The nexus of OS with diabetes has remained a matter of scientific debate since the 1980s (123), their association being explicitly propounded in the “common soil” hypothesis (124). OS plays a primary role in the pathogenesis of DM (125, 126) as manifested through many enzymatic, non-enzymatic, and mitochondrial mechanisms (127). Free radicals and peroxides are produced in large quantities in T2DM via glucose oxidation and non-enzymatic protein glycation, which can overwhelm antioxidant defense mechanisms leading to cellular damage (119), a phenomenon termed “hyperglycemic or metabolic memory” (128). Auto-oxidation of glucose generates hydroxyl radicals, while membrane-associated xanthine oxidase and nitric oxide synthase generate free radicals, ROS, and RNS (129). Accumulating these oxidants can result in the formation of lipid peroxidation products such as the highly hazardous malondialdehyde (MDA) and acrolein generated by free radical-driven peroxidation of polyunsaturated fatty acids like arachidonic and linoleic acids (113). Heightened MDA levels are typical of diseases with an OS component (130). Although ROS and free radicals are involved in the proinflammatory cytokine-mediated beta-cell injury, not all free radicals generated are implicated in their destruction, as in the NO case (131). High ROS levels also reduce the bioavailability of nitric oxide (NO) generated by endothelial cells, whose multifunctional signaling role is crucial to vascular integrity (132). The proper functioning of cellular antioxidant systems is the mainstay in limiting oxidative damage (129), especially in the context of DM (133).

DM harms the tissues by increasing the non-enzymatic formation of advanced glycation end products (AGEs) through the Maillard reaction and glucose auto-oxidation, leading to loss of protein function. The binding of AGEs to the receptor for AGE (RAGE) induces NADPH oxidase-1 to produce more ROS (134, 135) in addition to activating atherogenesis-promoting signal transduction mechanisms (136) like mitogen-activated protein kinase (MAPK) that further magnifies the inhibition of NO formation by AGEs (137). AGE-induced oxidative stress and the ensuing diabetic progression ultimately depend on the cellular balance between RAGE and AGER1; AGER1 binds and degrades AGEs while RAGE promotes oxidative stress and has a freer rein in chronic diabetes since AGER1 removal of AGE is suppressed (138).

Recent research suggests that mitochondria unavoidably generate significant ROS through oxidative phosphorylation (139). The ROS and RNS generated in mitochondria pass out into the cellular milieu, harming cytoplasmic organelles and damaging macromolecules (140), making DNA more vulnerable to mutagenesis (141). Critical cellular homeostases pathways such as autophagy and mitophagy that clear the cell of damaged macromolecules and organelles such as mitochondria and endoplasmic reticula appear less active in people with diabetes, although the mechanism is unknown (66). The diametrically opposed concept of mitohormesis proposes a dual dose-dependent action of ROS species, wherein, when produced by mitochondria in typically “small” amounts, they have a salubrious instead of a noxious effect. Some researchers proposed that DM may be a consequence of slowed-down mitochondrial machinery, which can restore health if restored to normal levels of superoxide production (142, 143). Intriguingly, a mitohormetic effect in animals has been demonstrated for the front-line antidiabetic metf (144). However, ambiguities remain since the studies did not consider the damaging effects of reductive stress that precedes oxidative stress in hyperglycemia on mitochondria and malfunctioning feedback mechanisms (142). Nonetheless, the importance of reducing the physiological factors that cause oxidative stress in conjunction with the use of natural antioxidant products, known as the “optimal redox” (OptRedox) approach, is being studied as a public health policy to stem the rising incidence of T2DM in global populations, particularly those of the very young (145). Instead of the common “detect and treat” medical practice of redox medicine, a more targeted use of inhibitors and agonists impacting oxidative stress linked biochemical pathways is hypothesized as a more successful therapeutic approach, while supporting empirical proof is currently a pipedream (108).

### NS as an antidiabetic agent

ROS levels rise throughout the progression of diabetes, and they are known to be implicated in the destruction of beta cells. TQ’s lack of effect on the transcription factor nuclear factor kappa-B (NF-κB), whose activation by OS is a prelude to diabetes, has not lessened its value as a potential therapeutic (146). It is still a powerful inhibitor of the inflammatory pathways that underpin autoimmune illnesses like T1DM, particularly those involving MAPKs (147, 148) and several *in vivo* and *in vitro* studies substantiate the antidiabetic efficacy of NS (38, 149–153). In comparison to fixed oil, the essential oil of NS is thought to have more effective antioxidant activity (154), and if stored appropriately, the antioxidant capacity of the NS oil rises over time despite having a lower TQ content than when it was first extracted (101). Both the NS volatile oil and its principal bioactive ingredient, TQ, are known to improve hyperglycemia and hyperlipidemia (83). In animal studies, NSO’s anti-hyperglycemic effect was equivalent to, if not superior to, metf, the principal hypoglycemic medicine now in use (155). Because this was a one-time trial, larger-scale research using more NS parameters may provide a clearer picture. NS has been linked to pancreatic islet regeneration, and the antidiabetic mechanism maybe due to NS’s ability to increase insulin secretion by boosting β-cell proliferation (156–158). In addition, NS extracts can decrease the body’s inflammatory and OS markers (159) as well as boost skeletal muscle glucose uptake and adenosine monophosphate-activated protein kinase (AMPK) activity (160). NS and TQ repress gluconeogenesis in the liver (161) by explicitly targeting the enzymes glucose-6-phosphatase and fructose-1, 6-biphosphatase (162) and also retard glucose absorption in the alimentary tract while enhancing glucose tolerance in rats (163). Lowering increased glucose uptake in diabetics by inhibiting the intestinal glucose transporter, sodium-glucose linked transporter 1 (SLGT1) through bioactive compounds represents a promising target for novel drug development (164). Controlling the postprandial glycemic spike in T2DM patients by blocking the digestive enzymes α-glucosidase and α-amylase with NS as opposed to clinical drugs that tax the gut physiology is also a feasible strategy (165). The feeding of NS extract decreased lipid peroxidation and increased antioxidant enzymes such as Superoxide dismutase (SOD), Catalase (CAT), and Glutathione peroxidase (GPx) in the organs of rats with chemically-induced diabetes (166, 167). The antidiabetic benefit of ground NSS via an antioxidant-based mechanism has been proven in large-scale clinical trials involving T2DM patients (168, 169). However, the same could not be said of NSO in such trials (170, 171) even though NSO supplementation causes a marked reduction in oxidative stress in healthy individuals (172). A host of confounding factors related to intervention methodology and the quality of the tested herbal product have been cited as the cause of this inconsistency (173). In a first-of-its-kind clinical trial comparing NS as monotherapy to metf in the treatment of diabetic patients, the former failed to achieve therapeutic outcomes (174). It could that the NS quantity was subtherapeutic (1,350 mg/day) since studies suggest that NS doses of less than 2 g are clinically inconsequential (168). On the basis of a meta-analysis of relevant clinical trials, the general consensus is that NS supplementation is an effective treatment for T2DM (175). Chronic T2DM marked by hyperglycemia-mediated insulin resistance remains a therapeutic challenge and the ameliorative impact of TQ has just lately come to light (176). TQ can also improve levels of insulin receptors improving insulin action, low levels of which are the cause of insulin resistance and type 2 DM (177). TQ can dramatically reverse the diabetes associated drop in Glut-2 levels (177), a transporter protein responsible for glucose transfer between the liver and blood and its reabsorption by the kidneys (178). By suppressing oxidative stress, reducing low-density lipoprotein (LDL), raising high-density lipoprotein (HDL-C), and lowering total blood cholesterol, TQ also mitigates cardiovascular complications such as atherosclerosis that accompany the course of diabetes (162, 179, 180).

It has been discovered that all forms of NS, including oil, water extracts, dried and crushed seed portions, show substantial hypoglycemic potential, particularly those based on aqueous extraction (181). Since this form of extraction gives the lowest TQ content (182), it implies the presence of active compounds in NS seeds other than TQ, of which there are over a hundred, many of which are unknown (183), but which may be equally beneficial in diabetes management (184). Multiple clinical trials (Table 1) and related studies (Table 2) evaluating different oral quantities advised for NSS, oils, and TQ have demonstrated that NS ingestion does not result in acute or chronic toxicity (219, 220) and is deemed safe among the hundreds of candidate medicinal plants and the oral antidiabetics currently available (76). However, few significant clinical trials on the safety and efficacy of NS have been conducted, highlighting the need for additional study (221). Some trial results, such as the one evaluating the suitability of NS supplementation for diabetic patients undergoing hemodialysis, have not yet been published (222). Even if unfavorable reactions in human subjects are uncommon, it is prudent to proceed with caution and prudence. There is some evidence linking NSS and essential oil to negative health effects in laboratory animals (223, 224). Consumption of NSS has been associated with inhibition of the drug-detoxifying enzymes cytochrome P450 2D6 (CYP2D6) and cytochrome P450 3A4 (CYP3A4), which raises the specter of unforeseen drug–drug interactions and prescription drug toxicity (225). Reaction to these enzymes is a key aspect of the protocols followed by worldwide drug regulatory authorities, including the FDA, when reviewing innovative drug candidates (226). TQ inhibits CYP2C19 and CYP3A4 *in vitro*; the latter is involved in the biotransformation of various oral antidiabetic drugs, a concern which must be investigated through *in vivo* studies (227).

**Table 1:** Clinical trials for NS and TQ supplementation on DM outcome.

Sr. No.	Dose and form of NS	Duration of treatment	Trial/Test design	Outcome	References
1	NS capsules: 500 mg/capsule used as 2 g/day	1 year	Randomized clinical trial 114 T2DM patients (18–60 Y) I: Control (charcoal-placebo) II: NS group	∙Significant decrease in FBG, HbA1c and TBAR ∙Significantly elevated TAC, SOD and GSX levels ∙Significantly low insulin resistance and upregulated β-cell activity ∙Long term NS supplementation is better than oral hypoglycemics in controlling glycemia and oxidative stress in T2DM patients	(169)
2	NS tea: 5 g/day	6 months	41 T2DM patients +25 healthy controls (identical in age) I: Control: NS tea II: NS tea + oral antidiabetic drug	∙Significant decrease in FBG, PPBG, and HbA 1c ∙Significant decrease in AST serum bilirubin, blood urea ALT, and serum creatinine ∙NS tea is recommended as supplemental antidiabetic therapy.	(185)
3	NS/capsule: 1 g, 2 g, and 3 g	3 months	Randomized controlled trial of 94 T2DM female patients (mean age range: 44.91–49.63 Y) I: NS capsule 1 g/day (n = 16) II: NS capsule 2 g/day (n = 18) III: NS capsule 3 g/day (n = 17)	∙NS (1 g/day) minor improvement in all the measured parameters from the baseline. ∙NS (2-3 g/day) significant reductions in FBG, 2hPG, and HbA1 ∙No significant change in BW. ∙Reduction in insulin resistance (*p* < 0.01) ∙Increase in β-cell function (*p* < 0.02) after 12 weeks of treatment. No adverse effects on renal and hepatic functions with either dose	(168)
4	NSS: 250 Mg*trigonella foenum-graecum*: 250 mg	3 months	100 T2DM patients (30 to >40 Y) Male and female I: Control (Glibenclamide) II: Intervention (NS + *Trigonella foenum-graecum* seeds + Glibenclamide)	∙Significant increase in serum HDL levels ∙No significant change in serum creatinine and triglyceride levels	(186)
5	NSO:1,350 mg/day	3 months	66 newly diagnosed T2DM patients (≤6 months) (18-60Y) I: Metf II: NS oil capsule,	∙NS was inferior to Metf in glycemic control (in lowering FBG, 2 h pp., and A1C or increasing %B) ∙NS was comparable to Metf in significantly lowering weight, WC, and BMI. ∙NS was comparable to Metf in its effects on fasting insulin, %S, IR, ALT, TC, LDL, HDL, TG, and TAC. ∙Metf showed a significant increase in AST and creatinine compared to NSO.	(174)
6	TQ: 50 mg/kg	90 days	60 T2DM I: 1 metf +1 TQ II: 1 metf +2 TQ III: 1 metf	∙Glycated hemoglobin (HbA1c) levels decrease after 3 months of TQ intake. ∙A more significant reduction in FBG and postprandial blood glucose was also observed in TQ receiving groups compared to metf alone	(17)
7	NSO: 3 g/day	12 weeks	72 T2DM patients (30–60 Y) I: Treatment (NSO) II: Placebo (sunflower oil)	∙Insignificant BW and BMI reduction ∙Dietary intake in both groups changed compared to baseline. ∙Significant changes in FBS, HbA1c, TG, and LDL-c ∙Insulin level and insulin resistance decreased and ∙Insignificant increase in HDL-c	(170)
8	NS soft gel capsules: 500 mg Twice/day	8 weeks	A randomized controlled 43 (23 women, 20 men) T2DM participants (30–60 Y) I: NSO II: sunflower oil	∙NSO significantly decreased FBS, HbA1c, total cholesterol, TG, LDL-c, BMI, waist circumference, SBP, and DBP. ∙No significant change in HOMA-IR and HDL-c	(187)
9	NSO: 2.5 ml twice/day	6 weeks	60 patients (50 males and 10 females) with obesity, diabetes, dyslipidemia I: Met + atorvastat II: Met + atorvastat + NSO	∙Significant improvement in total cholesterol, LDL-c, and FBG	(188)
10	NSO equivalent to 0.7 g of seed/day	40 days	41 T2DM patients 1^st^ 40 days NS treatment, following 40 days placebo treatment	∙Significant decrease in FBG and increase in insulin and AST levels ∙No changes in platelet count, total leukocyte count, and ALT blood urea comparable to baseline levels.	(189)
	NSS	40 days	Male + female T2DM patients (30-60Y) 1^st^ 40 days NS treatment, following 40 days placebo	∙Improved levels of BGL, INS, and lipids ∙Decreased fasting blood glucose, TC, LDLc, TG, HDL	(190)
11	NS: 1 g, 2 g and 3 g	30 days	45 diabetic patients I: NS 1 g (glucose <180 mg/dL) II: NS 2 g (glucose 180–220 mg/dL) III: NS 3 g (glucose >220 mg/dL)	∙Significant improvement in blood glucose (2 g NS shows better performance) ∙Negligible improvement of lipid profile in all groups.	(191)
12	NSO: 5 ml/daily	3 months	70 T2DM patients (30 males, 40 females) I: NSO II: Mineral oil (placebo)	∙Significant decrease in the blood levels of fasting and 2 h postprandial glucose and HbA1c and BMI ∙No side effects	(192)
Prediabetic/metabolic syndrome					
13	NSO capsule: 450 mg twice /day	6 months	Open-label, randomized prospective comparative 117 prediabetic patients (18–65 Y) I: LM group, a calorie-restricted diet with moderate exercise II: Metf group, Metf tablet 500 mg/day for initial 2 weeks, then same was given twice/day III: NS soft gelatin capsules containing 450 mg NSO twice daily Group II and III did not follow a lifestyle management program (LM)	∙NS was statistically like Metf in improving anthropometric glycemic parameters and SIRT1 gene expression. ∙NS improved lipid panel and suppressed inflammation ∙Significantly reduced TNF-α and Castelli risk index-I ∙NS may represent a promising intervention for obese prediabetic subjects	(193)
14	NS powder: 1.5 g/day In combination, NS: 900 mg/day	8 weeks	Double-blind, randomized, 250 healthy male (MetS participants) (44 ± 13.3 Y) I: Powdered NSS II: NS powder + Turmeric powder III: Placebo of Ispaghul husk	Week 4: ∙Showed improvement in BMI, WC, and BF%. ∙The combination improved all parameters except HDL-c with lower FBG and LDL-c compared to placebo. Week 8: ∙Reduced lipids and FBG, ∙Combination group with a 60% dose of the individual herbs showed an improvement in all parameters from baseline. ∙Reduced BF%, FBG, cholesterol, TG, LDL-cholesterol, and CRP, but raised HDL-cholesterol.	(194)
15	NSO 500 mg/day	8 weeks	80 metS patients (52 male, 38 females) 20–70 Y (majority 40-60Y) I: Met + Astorvastatin + Aspirin II: NSO + Aspirin	∙NS significantly lowered FBG, PPBG and HbA1c after 8 weeks. The NS group showed significant improvement in FBG, PPBG, HbA1c, and LDL cholesterol. ∙NS is safe and an effective remedy for patients with metabolic syndrome	(195)
Safety/toxicity studies					
16	TQ: 10, 20, 100, 200, 400 mg capsules	1 to 20 weeks	18 adult patients with solid tumors or hematological malignancies (at least 18 Y) with an Eastern cooperative oncology group performance status (ECOG) of ≤2; received TQ orally at a starting dose of 3, 7, or 10 mg/kg/day. Dose escalation was done using a modified Fibonacci design.	∙No side effects or systemic toxicities were reported, and the maximum tolerated dose (MDT) was not identified. ∙No anti-cancer effects were observed. ∙Tolerable oral TQ dose ranging from 75 mg/day to 2,600 mg/day	(196)
17	TQ: 200 mg capsules	90 days	70 healthy adult volunteers (phase I randomized, double-blinded, placebo-controlled trial clinical trial) Each participant received a single daily dose of 200 mg/day, 10–20 min before bedtime.	∙No significant alterations in the hematological parameters ∙No significant changes in the biochemical parameters of liver function ∙(ALT, AST, ALP), renal function (serum creatinine and urea) ∙5% TQ v/v in NS oil at a dose of 200 mg/adult/day is safe for human consumption and ought to be clinically evaluated for various health related pharmacological activities.	(197)

metS, metabolic syndrome; T2DM, type II diabetes mellitus; NS, *Nigella sativa*; FBG, fasting blood glucose; FBS, fasting blood sugar; HbA1c, glycosylated hemoglobin; TG, triglyceride; LDL-c, low-density lipoprotein cholesterol; HDL-c, high-density lipoprotein cholesterol; SBP, systolic blood pressure; DBP, diastolic blood pressure; HOMA, homeostatic model assessment of insulin resistance; BMI, body mass index; WC, waist circumference; SOD, superoxide dismutase;AST, aspartate aminotransferase; ALT, alanine Aminotransferase; ALP, alkaline phosphatase; BUN, blood urea nitrogen.

**Table 2:** Animal studies done in the last 5 years for assessing NS and TQ safety and dosage.

Sr. No.	Dose and form of NS	Duration of treatment	Trial/Test design	Outcome	References
1	TQ: 50 mg/kg in 0.1% DMSO	12 weeks (84 days)	60 male Wistar rats (age?) STZ-induced DM rats I: Healthy control II: DM untreated control II: DM TQ treated III: DM TQ-vehicle control Treatment via gastric gavage	Reduction in NO and MDA levels in testicular tissue ∙Exerted a protective effect against reproductive dysfunction induced by diabetes	(198)
2	NS powder: 300 mg/kg	8 weeks (56 days)	24 Albino rats (sex?) (age?) 8 weeks high-fat diet (HFD) I: Control II: HFD untreated control III: HFD virgin olive oil treated IV: HFD NS-treated Treatment via intragastric intubation	∙Reduce the serum lipid profile, TC, TG, LDL, HDL, BGL and amylase ∙Significant increase in INS levels ∙Regeneration of the exocrine and endocrine parts of the pancreatic tissues	(199)
3	TQ: 80 mg/kg	7 weeks(49 days)	50 white male albino rats (*Rattus norvegicus*) (6–7 weeks) STZ-induced DM I: Control II: DM control III: DM TQ-treated IV: DM Met-treated V: DM Met+TQ-treated Treatment via p.o.	∙Decreases the MDA levels and up-regulated the expression of Glut-2 ∙Enhance the antidiabetic activity of MET in STZ-induced diabetic rats	(200)
4	NS_1: 100 mg/kg NS_2: 200 mg/kg NS_4: 400 mg/kg	6 weeks (42 days)	70 male Wistar rats (10 weeks) STZ induced DM i.p. I: Control II: DM control III: DM NS_1-treated IV: DM NS_2-treated V: DM NS_4-treated Treatment via gavage	∙Reduced serum glucose, lipids and improved AIP (Atherogenic index of plasma) ∙Significantly increased eNOS (endothelial nitric oxide synthase) ∙Decreased VCAM-1 and LOX-1 expression	(149)
5	NSO: (91 mg/100 ml) 1. 0.5 ml 2. 1 ml 3. 1.5 ml	40 days	30 Laboratory bred male albino Wistar rats (age?) STZ-induce DM i.p. I: DM control II: DM Met-treated III: DM NS-treated (1) IV: DM NS-treated (2) V: DM NS-treated (3) Treatment via p.o.	∙Significant reduction in BGL ∙Partial regeneration of β islet cells of the pancreas by 1.5 ml of NS	(201)
6	NSSP (NS seed polysaccharides) (0.1 ml/10 g) High dose: 140 mg/kg Med dose: 70 mg/kg Low dose: 35 mg/kg	4 weeks	60 male pathogen-free Kunming mice (4 weeks), 4 weeks on a high-fat diet (HFD) STZ-induced diabetes I: Control II: DM Met-treated III: DM NSSP high dose treated IV: DM NSSP med-dose treated V: DM NSSP low dose-treated Administration via intragastric tubing	∙High-dose NSSP could significantly lower the levels of FBG, GSP, TG, TC, LDLc, MDA, TNF-α, IL-6, and IL-1β, and ∙Significantly increased INS, HDLc, T-AOC, SOD, CAT, p-AKT and GLUT4 ∙NSSP could improve the abnormal state of diabetic mice by regulating the PI3K/AKT signaling pathway with simultaneous changes in the gut microbiota profile.	(202)
7	TQ: 50 mg/kg BW	4 weeks (28 days)	18 male Sprague–Dawley rats (age?) STZ-induced DM I: Control II: DM III: DM TQ-treatment Treatment via gastric gavage	∙Significantly lower levels of HbA1c, lipid peroxidase, and NO ∙Higher TAC ∙Attenuated the effect of STZ-induced diabetic nephropathy ∙TQ adjusts glycemic control and reduces oxidative stress without significant damaging effects on renal function.	(203)
8	TQ in corn oil TQ-10: 10 mg/kg TQ-20: 20 mg/kg	21 days	40 male Wistar rats (age?) STZ-induced T2DM I: Control II: DM untreated III: DM TQ-10 treated IV: DM TQ-20 treated V: DM TQ-10 + fluoxetine treated VI: DM TQ-10 + fluoxetine treated Treatment via p.o.	∙TQ decrease in BGL, no further significant change was recorded with TQ + fluoxetine treatment. ∙Significantly decreased immobility time ∙Increased latency to immobility and locomotor activity ∙TQ and fluoxetine combination reduced TBARS level and increased GSH content but did not affect antioxidant enzyme activities. ∙Reduction in inflammatory markers (IL-1b, IL-6 and TNF-a) TQ + fluoxetine can be used to control depression	(204)
9	TQ: 50 mg/kg	21 days	Male ICR (CD1) mice (Envigo, IN, USA) (8–9 weeks) STZ induced DM I: DM met treated II: DM metf + TQ treated Treatment via p.o.	TQ showed a significant decrease in BGL compared to metf	(17)
10	TQ: 20 mg/kg 40 mg/kg 80 mg/kg	21 days	Wistar female albino (age?) Nicotinamide + STZ induced T2DM i.p I: Vehicle control II: DM control III: DM Met treated IV: DM Met-NCs treated V: DM TQ-20 treated VI: DM TQ-40 treated VII: DM TQ-80 treated VIII: DM TQ NCs-20 treated IX: DM TQ NCs-40 treated X: DM TQ NCs-80 treated XI: DM blank NCs Treatment via p.o.	∙NCs showed a sustained release profile as compared to their pure forms. ∙TQ or Met and their NCs significantly decreased BGL and HbA_1c_ ∙improved the lipid profile ∙TQ-loaded NCs produced a dose-dependent antihyperglycemic effect comparable to TQ and Met. TQ NCs (containing half of the doses of TQ) produced a better antihyperglycemic effect in T2DM rats than TQ alone.	(205)
11	TQ: 10 mg/kg TQ loaded NFs: 10 mg/kg	21 days	60 Wistar female albino rats (age?) Nicotinamide-STZ induced DM i.p. I: Control d. H2O II: Control oleic acid III: Diabetic control IV: DM Met treated V: DM GL treated VI: DM TQ treated VII: DM GL + TQ treated VIII: DM GL-NFs treated IX: DM TQ-NFs treated X: DM GL + TQ-NFs treated	∙Significant decreases in BGL and HbA_1c_ ∙Significant improvements in BW and lipid profile Synergistic effect of combined NFs, leading to enhanced absorption of NFs and lesser cytotoxicity than pure bioactive compounds	(206)
12	TQ: 10 mg/kg 20 mg/kg	14 days	30 Wistar rats (8 weeks) STZ-indued T2DM in 4-week HFD I: Control II: DM control III: DM TQ-10 treated IV: DM TQ-10 treated V: TQ control Treatment via p.o.	∙Significantly prevented hyperglycemia, hyperinsulinemia, hyperlipidemia, INS resistance, and inhibited DPP-IV ∙An alternative natural drug in the management of hyperglycemia-induced INS resistance	(176)
13	TQ?	1 h	Male Sprague–Dawley rats (age?) STZ induced T1DM 2-week daily pretreatment (via oral gavage) of T1DM rats with Sitagliptin I: TQ treatment in sitagliptin-pretreated II: TQ treatment in non-sitagliptin-pretreated Additionally, varying doses of exendin 9–39 were pretreated 30 min before TQ in DM with or without pretreatment with sitagliptin. Treatment via i.p.	∙The direct effect of TQ on imidazoline receptors (I-Rs) was identified in CHO-K1 cells overexpressing imidazoline receptors (I-Rs). ∙Enhances GLP-1 secretion by intestinal NCI-H716 cells TQ may promote GLP-1 secretion through I-R activation to reduce hyperglycemia.	(207)
14	Methanol NS Plant extract: 500 mg/kg BW	120 min for glucose 240 min for sucrose	40 males + female Long Evan rats (age?) STZ induced T2DM	∙The extract reduced postprandial glucose, ∙Improved glucose (2.5 g/kg, BW) tolerance in rats. ∙Significant improvement in GI motility ∙Reduced disaccharidase enzyme activity in fasting rats. ∙Potential hypoglycemic activity ∙Significantly improved INS secretion from isolated rat islets. Generate postprandial anti-hyperglycemic activity in T2DM animal models via reducing or delaying carbohydrate digestion and absorption in the gut and improving INS secretion in response to the plasma glucose.	(208)
15	Ethyl acetate fraction of Ethanolic NS plant extract: 200 mg/kg, 500 mg/kg and 1,000 mg/kg BW		25 male rats (age? Breed?) Alloxan induced T2DM	∙Reduced blood glucose levels	(209)
16	Hydroalcoholic extract of NS seed: 200 mg/kg and 400 mg/kg	Oral administration for 4 weeks	24 male Wistar rats (age?) STZ induced T2DM	∙Reducing effect on FBS and oxidative biomarkers ∙Increases serum insulin levels	
17	Ethanolic extract of NS seed, using 20 and 40% wt/wt of feed	Oral administration for 15 days	35 Wistar albino rats (age?) (gender?) Alloxan monohydrate induced T2DM	∙Significant decrease in blood glucose levels ∙Significant antioxidant activity (elevated SOD levels)	(210)
18	Methanolic extract of NS seed and NS oil; 2.5 ml/kg/day	Oral administration for 24 days	15 male rabbits (age?) Alloxan (150 m/kg) induced T2DM	∙Both NSS methanolic extract and NSO were significantly hypoglycemic ∙NSO was more effective than methanolic extract of NSS in reducing serum catalase, ascorbic acid and bilirubin	(211)
19	Ethanolic extract of NS seed (100 mg/kg/BW) and TQ (10 mg/kg/BW)	Oral administration for 28 days	28 male Wistar rats (age?) STZ (90 mg/kg/BW) induced T2DM	∙significant decrease in blood glucose, urea, creatinine, uric acid, total protein, total cholesterol, low-density lipoprotein, and very low-density lipoprotein, while high-density lipoprotein was increased. ∙Hepatic enzymes, alanine transaminase, aspartate aminotransferase, and alkaline phosphate were also normalized. ∙significantly increased body weight.	(212)
20	Ethanolic extract of NS seeds; 300 mg/kg/BW and 600 mg/kg/BW	Oral administration for 7 days	Male Wistar rats (200–250 g BW), number? age? gender? STZ (50 mg/kg/BW) induced T2DM	∙Significant reduction in blood glucose, total cholesterol, triglycerides, ∙VLDL and non-HDL cholesterol comparable to metformin	(213)
21	NS oil; 2.5 ml/kg/BW	Oral administration for 56 days	30 Male Wistar rats (age?) STZ (45 mg/kg/BW) induced T2DM	∙NS oil significantly normalize blood urea ∙Significantly nephroprotective and anti-DM	(214)
Safety/toxicity					
22	NS powder: 3 g/kg/day NSO: 2 g/kg/day NS ethanol extract: 0.5 g/kg/day	60 days	50 male Sprague–Dawley rats (age?) Cisplatin-induced nephrotoxicity I: Healthy control II: Diseased positive control (d. H2O) III: NS powder treatment IV: NSO treatment V: NS extract treatment Treatment via stomach tube	∙Reduced serum levels of urea, creatinine, and K ∙Significant increase of Na, Na/K, vitamin D, nutritional markers, and antioxidant enzymes. All forms of NS contain potent bioactive components that help in cisplatin-induced renal toxicity in rats.	(93)
23	Aq. NS extract: 2 g/kg 6.4 g/kg 21 g/kg 33 g/kg 60 g/kg BW	6 weeks (42 days)	Subacute toxicity Female *Mus musculus* mice (6–8 weeks) I: Control II: NS 2 g/kg III: NS 6.4 g/kg IV: NS 21 g/kg V: NS 33 g/kg VI: NS 60 g/kg Antidiabetic effect female Wistar rats*, Rattus norvegicus*, Alloxan-induced DM i.p I: Control II: DM control III: DM NSE treated (2 g/kg) Treatment via an esophageal probe	∙Aq. NS extract showed no variation in urea and albumin following the five doses administered ∙Significantly decreased glycemia, TG, TC, LDLc, and TBARS ∙Restored insulinemia and a significant increase in HDLc. ∙Liver indicated a decrease in lipids and possible glycogenesis.	(181)
24	NS extracts: 1 g/kg 3 g/kg 5 g/kg 7 g/kg 10 g/kg BW NS fractions: 0.1 g/kg 0.3 g/kg 0.5 g/kg 0.7 g/kg	Signs of toxicity were observed after 2 h and every 24 h till 14 days	30 male + female Swiss albino mice (age?) I: Control II: NSE dose III: NSF dose Treatment via p.o.	∙NS extracts were nontoxic up to a concentration of 10 g/kg.	(165)
25	NSOCS1: 1 ml/kg NSOCS2: 2 ml/kg	8 days	Healthy adult male+female albino rats (age?) I: Control II: Negative control: CMC p.o. + colistin III: NSOCS1: NSO + colistin IV: NSOCS2: NSO + colistin	∙Dose-dependent improvement in tubular damage and reduced biochemical alteration. ∙NSO reduces colistin sulfate-induced nephrotoxicity, especially in a higher dose of 2 ml/kg.	(94)
26	NSO: 4 ml/kg	24–48 h	24 female Wistar-albino rats (age?) I: Control group II: NSO 48 before, saline 24 h before sacrifice III: Saline 48 h before, carboplatin 24 h before. IV: NSO 48 h before, carboplatin 24 h before Treatment via i.p.	∙Reduce the degeneration in hepatocytes, fiber distribution, and density around the central vein and portal space ∙Hepatocyte cords preserved integrity, partial degeneration in hepatocytes, and decreased collagen fiber distribution around the central vein. ∙Insignificant lower apoptosis	(215)
27	TQ: 15 mg/kg	24 h	36 healthy male albino rats (age?) I: Control II: Acetaminophen (APAP) III: N-acetylcysteine (NAC) IV: α-Lipoic acid (ALA) V: TQ VI: ALA+TQ 3-doses, 1^st^ before 24 h, 2^nd^ after 2 h, and 3^rd^ after 12 h of APAP dose. Treatment via p.o.	∙Treatment with all antioxidants ameliorated most of the altered parameters ∙Treatment with the combination of ALA and TQ was the most effective therapy in the attenuation of liver injury ∙Marked improvement in hepatic degeneration ∙Natural antioxidants such as ALA and TQ may be considered as a potential antidote in combating liver injury induced by APAP	(216)
28	NSO-1: 1 ml/kg NSO-2: 2 ml/kg NSO-4: 4 ml/kg BW Subacute NSO: 4 ml/kg BW	360 min Subacute inflammation: 168 h	50 white female rats (Wistar-Bratislava) (age?) [Same animals were used for acute and chronic models with a wash-up of 2 weeks] Carrageenan-induced acute inflammation I: Control (saline) II: Positive control (Diclofenac sodium) i.p. III: NSO-1 IV: NSO-2 V: NSO-4 Oral route Freund’s adjuvant-induced sub-acute inflammation I: Control (saline) II: Posivitve control (Diclofenac sodium) III: NSO-pre (7-day before FA) IV: NSO-treat (7 days after FA) V: NSO-adj (NS + Diclo) (7 days after FA)	Significant inhibitory effect of NSO on paw edema in all three doses In the acute phase, 1.5 h after administration, NSO (2 and 4 ml/kg) showed an anti-inflammatory effect comparable with diclofenac. In the sub-acute administration, ∙NSO had no anti-inflammatory effect. ∙Analgesic effect was observed only in the sub-acute inflammation ∙An antioxidant effect through the reduction of MDA and GSSG	(217)
29	NSO-0.6% and 5% (w/w) TQ	90 days, single dose, acute and subchronic repeat dose	Acute toxicity study: 3 adult female Wistar rats given 5, 50, 300 and 2,000 mg/kb BW Subchronic repeated dose toxicity study: 5 male and 5 female adults Wistar rats per group as follows: 1 Control II: NSO 94 mg/kg BW (5% TQ) III: NSO 47 mg/kg BW (2.5% TQ) IV NSO 9.4 mg/kg BW (0.5% TQ)	Black cumin oil containing 5% (w/w) of TQ content was found to have a “no-observed-adverse-effect-level” NOAEL of 0.1 ml/ kg or 94 mg/kg b. wt. in rodents, which also corresponds to a dose of 5 mg of TQ/kg b. wt. From this study, the safe human dosage may be derived as not more than 900 mg/kg b. wt. of BCO-5/day or 50 mg of TQ/adult/day.	(218)

NS, *Nigella sativa*; NSE, NS extract; NSFO, NS fixed oil; NSEO, NS essential oil; TQ, thymoquinone; DM, diabetes mellitus; STZ, streptozotocin; BW, body weight; CAT, catalase; GPx, glutathione peroxidase; GST, glutathione-S-transferase; GSH, reduced glutathione; NCs, nanocapsules; NFs, nano formulations; CPK, creatine phosphokinase; MDA, malondialdehyde; MDH, malate dehydrogenase; HbA1c, glycated hemoglobin; BGL, blood glucose level; p.o., per os; i.p., intraperitoneal; NO, nitric oxide; TC, total cholesterol; INS, insulin; TAC, total antioxidant capacity; GSP, glycosylated serum protein; GSSG, oxidized glutathione;?, details unclear.

### Hepatoprotective and lipid-lowering effects of NS

Due to the features of several new medications, the necessity for hepatoprotection is becoming an increasing concern (228). Acute hepatotoxicity can lead to liver cancer (229), and almost half of drug-induced hepatoxicity cases were attributed to acetaminophen/paracetamol overdosing (230). The liver is the primary site of drug detoxification and is particularly vulnerable to OS (231). NS, because of its protective activity against an array of natural and synthetic toxins (232), including xenobiotics (233), and because of its relative safety and potent antioxidant and anti-inflammatory effects, is ideal for reducing the side effects of neoadjuvant therapy of the cancerous liver before ablative surgeries (228). NSS extracts stabilized lipopolysaccharide-induced hepatotoxicity by normalizing levels of aspartate aminotransferase (AST), Alanine aminotransferase (ALT), and Alkaline phosphatase (ALP) (234). Similarly, NSO effectively raised antioxidant enzyme levels and improved liver function in malathion-induced liver dysfunction (235) and hypervitaminosis (236). TQ also normalized hepatic OS and lowered cholesterol levels, which were elevated due to a cholesterol-rich diet (237). Research has demonstrated that TQ can normalize the amounts of the liver enzymes such as oxidized glutathione (GSSG), SOD, and MDA and enhance reduced glutathione (GSH), essentially protecting against oxidant damage to the liver (238). NSS also augmented the hepatoprotective effect by enhancing CAT, and GPx activity (215, 239). Liver damage due to therapeutic drug overdose is a growing health concern (240). Presently, N-acetylcysteine is the sole clinical intervention for acetaminophen overdose, but its drawbacks such as poor bioavailability, high costs, and side effects warrant exploration for a new, natural curative (221, 241–243). Acetaminophen (APAP)-related hepatotoxicity was effectively countered in experimental rats receiving a combination of α-Lipoic acid (ALA) and TQ as assessed by a decrease in ALT and ALP function and down-regulation of cyclooxygenase-2 (COX-2) and vascular endothelial growth factor (VEGFR1, flt-1) expression (216). Another study suggested that TQ’s mechanism for attenuating APAP-induced acute liver injury involved inhibition of the entire MAPK family with simultaneous activation of the AMPK pathway (221). High doses of NS supplements over prolonged periods can reduce ALP levels substantially, but because of the imprecision of NS’s dose, duration and effect on liver parameters, its approval as a treatment for liver ailments has remained elusive (244).

### Antioxidant activity of NS and TQ

Since the 1990s, the notion of antioxidants has been in the public eye, and their role in disease prevention a subject of interest. They have been defined as “any substance able to eliminate ROS and derivatives (RNS, or reactive sulfur species, RSS), directly or indirectly, acting as an antioxidant defense regulator, or reactive species production inhibitor” (245). Using antioxidants to complement diabetes therapy has gained much prominence, and many compounds of plant origin and vitamins have been scrutinized as possible candidates, each with its challenges and shortcomings (246–249). NS essential oil has been markedly better at radical scavenging than many commercially available synthetic antioxidants (250). Recent work has shown that TQ, carvacrol, t-anethole, 4-terpineol, tannins, flavonoids, and alkaloids contribute to the radical scavenging properties of NSS with TQ and nigellone accounting for the majority of the activity (251). NS sustains the cellular microenvironment by increasing the body’s antioxidant enzymes, SOD, GPx, and CAT, and enhancing ROS scavenging capacity (169) by increasing vitamin C and E levels (252). In addition to TQ, NS contains other antioxidants such as flavonoids, phenolics, ascorbic acids, and tocopherols (253). Because of its antioxidant capacity, TQ decreases tissue MDA levels, prevents DNA damage, reduces mitochondrial vacuolization and fragmentation, and maintains pancreatic β-cell integrity (252). Lipid peroxidation is a marker of significant stress, and TQ can reduce lipid peroxidation by its robust scavenging of ROS (254). TQ can be reduced to thymohydroquinone under normal intracellular physiological conditions, and to the pro-oxidant semiquinone under pathological conditions with excess of metal ions (255, 256). Based on examination of its molecular structure, the claim that reduced thymohydroquinone possesses any radical scavenging activity, let alone exceeding that of TQ, has been questioned (257). The non-enzymatic binding of TQ with intracellular antioxidants, GSH, NADH, and NADPH results in moieties whose scavenging potency far exceeds that of the free TQ and is at par with Trolox, a powerful antioxidant and vitamin E analog (258, 259). TQ can also be reduced to dihydro-thymoquinone by DT diaphorase, which induces oxidative stress from ROS, causing cytotoxicity and DNA damage (100). Dihydro-thymoquinone becomes a concern in specific circumstances, such as cancer, where DT diaphorase levels are elevated, or in animal trials where large quantities of TQ are administered (260). TQ’s lipophilic properties are similar to those of the mitochondrial electron transporter ubiquinone, and more research is needed to determine the precise link between thymohydroquinone and mitochondria (261).

**Figure 2:**
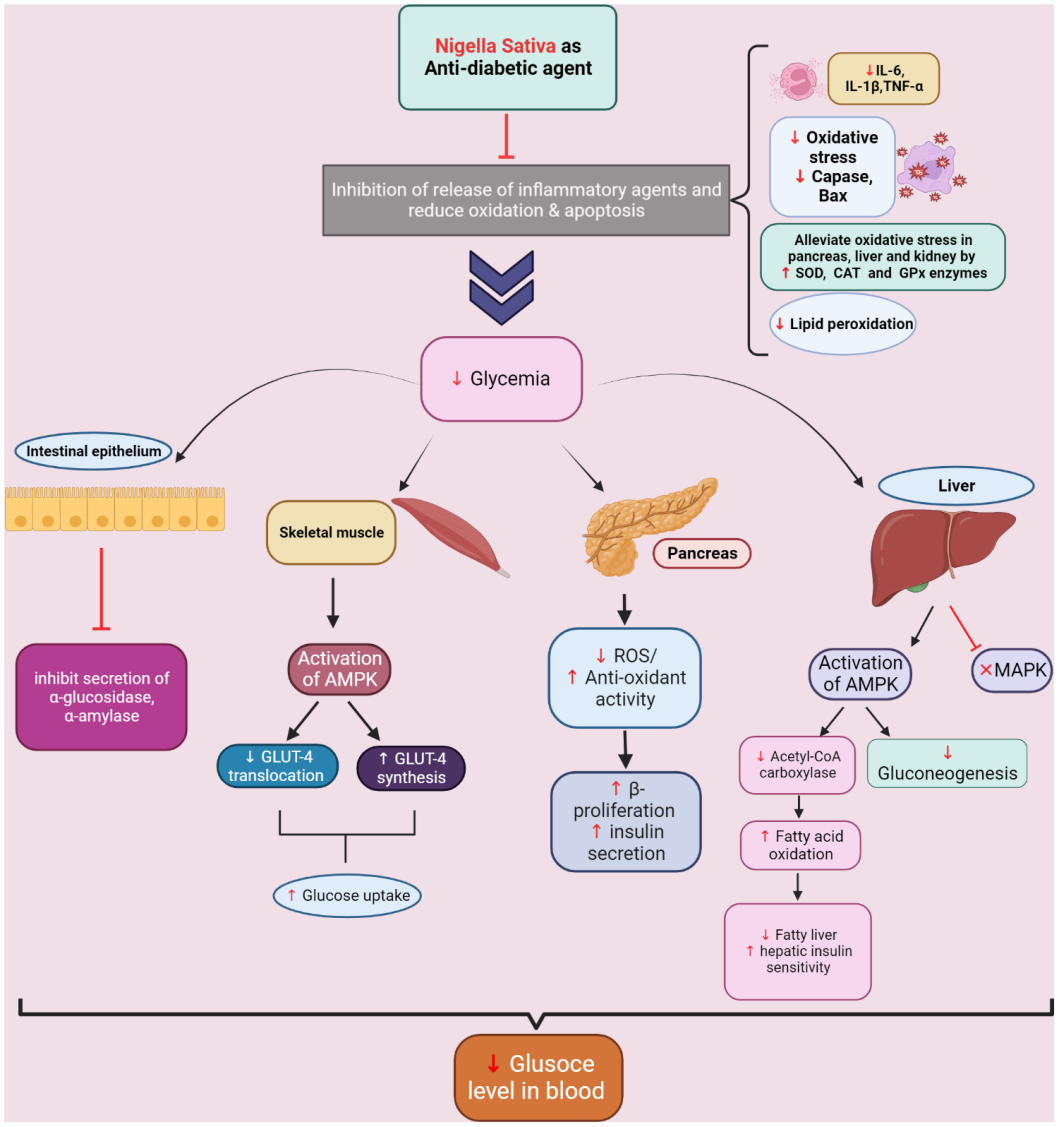
Antidiabetic action of *Nigella sativa.*

NS and TQ improve insulin sensitivity by increasing MAPK pathway activation, muscle GLUT-4 levels, which helps to gradually normalize glycemia (Figure 2). TQ also in a dose-dependent manner inhibits COX and lipoxygenase (LOX) enzyme activities, consequently blocking the synthesis of inflammatory mediators, prostaglandins, thromboxane, and leukotrienes and reducing joint inflammation (262). TQ by suppressing pro-inflammatory cytokines, interleukin-1β (IL-1β), and tumor necrosis factor-alpha (TNF-α), as well as interferon-gamma (IFN-γ), and interleukin-6 (IL-6), mitigates disease severity. TQ also inhibits NO production from activated cells and macrophages, increasing inflammatory responses and promoting apoptosis (254). The antioxidant properties of NS and TQ were responsible for reversing streptozotocin-induced modifications in creatine kinase-MB and brain monoamines (263). It has been demonstrated that NS promotes AMPK in the liver and muscles, resulting in antioxidant and health-protective effects. Synthetic AGE inhibitors have largely failed to combat hyperglycemic and OS-related AGEs due to side effects, hence attention has switched to natural plant-based extracts (264). NS represents a promising natural candidate since both TQ and NSS extract have been found to inhibit AGE formation *in vitro* (265, 266). However, the NS-mediated AGE inhibition mechanism remains largely unknown (267). Recent computational studies have made significant advancements in our mechanistic knowledge of TQ’s anti-glycation action on the eye lens crystallin proteins and its therapeutic promise in reducing DM-related ocular cataract (268). Furthermore, nothing is known about how NS and TQ interact with novel AGE forms such as melibiose-derived (MAGE), which is detected in high amounts in diabetics with microangiopathy (269). Because of their antioxidant content, both NSS and its alcohol or aqueous extract have effectively reduced diabetes-induced cytotoxicity *in vitro* (270, 271) and diabetic lab animals (272). However, it must be borne in mind that solvent extracts of Nigella seeds carry an array of antioxidants that can complicate establishing causality (90). Exogenous antioxidant supplementation for diabetic patients has some advantages, but the strategy was initially disregarded due to worries about a lack of clinical evidence and potential side effects (273), and it is still not used as a clinical strategy today (274), despite positive results from clinical trials using antioxidant supplementation for particular diabetic complications (274–276). It is broadly understood that any potential new antioxidant therapy for DM must target the disease and simultaneously effectively prevent the associated vascular complications (247).

### TQ and its importance

Using the isolated and purified active principle of medicinal plants instead of the crude extract is advantageous because it circumvents the variability in the content of the bioactive compound in the natural materials and losses due to processing and preparation. Avoiding potential interactions among the constituents using a purified preparation allows for more accurate and reproducible dosage and better analytical assaying of safety and efficacy (36, 277). Several compounds derived from NS have therapeutic value, but none comes close to TQ as the main bioactive component with the most diverse pharmacological benefits (83, 278). It has been argued that the effectiveness of NS fractions in lowering glycemia and serum lipid levels might be a function of their TQ content, with the volatile oil outperforming the others (175). TQ is a monoterpene benzoquinone compound synthesized in plants from γ-terpinene during secondary metabolism (279). Reportedly first isolated from NSS in the 1960s (73, 280), it is a yellow crystalline substance chemically known as 2-isopropyl-5-methylbenzo 1,4 quinone having a molar mass of 164.20 g/mol with a molecular formula of C_10_H_12_O_2_ (281) and CASRN: 490–91. Its tautomerism, where only the keto form of the molecule is thought to be pharmacologically active (162, 281), is disputed, and the reduced form of TQ has been misinterpreted as the enol form (282).

TQ has been shown in animal studies to effectively treat OS-related diseases with few or no side effects (36). It has been shown to have antioxidant, anti-inflammatory, antineoplastic, antimicrobial, analgesic, hypoglycemic, antihypertensive, and hepatoprotective properties (162, 254, 283, 284). TQ’s oxidant-scavenging prowess has been ascribed to molecular quinone and the ease with which it passes through cell membranes to reach intracellular targets (285). TQ represents a relatively new class of compounds with antioxidant ability in CH bonds rather than phenolic OH groups (171, 286). The TQ molecule has specific CH groups whose bond dissociation values of the hydrogen atom transfer mechanism impart a free radical-based antioxidant activity comparable to potent antioxidants like ascorbic and gallic acids (171).

Depending on the cellular and physiological milieu, TQ can undergo both enzymatic and non-enzymatic redox reactions to generate either pro-oxidants (semiquinone) or antioxidants (thymohydroquinone); the former is associated with ROS generation, while the latter exert radical-scavenging activity (278). Animal studies have shown that TQ synergizes with metf to markedly reduce serum glucose, HbA1c, MDA, and TAC levels, which neither of them could do as well individually, substantiating its combinatorial role in conventional drug therapy (177). However, clinical trials using such combinations have reported minor health concerns that merit further investigation (287).

TQ has significant pharmacological and pharmacokinetic potential to be a strong drug candidate, as reflected by its compliance with Lipinski’s “rule of five” (176, 288). One of the main problems in testing TQ in clinical trials has been a lack of standardized protocols to ensure uniform quality and dosage (182) (Table 1). Its high hydrophobicity, time-dependent aqueous solubility, aversion to alkaline pH, and significant photo-and thermolability have challenged pharmaceutical formulations (289). It is also poorly bioavailable and vulnerable to transformation by liver enzymes upon oral intake, which necessitated the development of a version encapsulated in nanoparticles that have proven more effective than the non-encapsulated natural TQ as an anti-glycemic drug (103, 205, 290, 291). Recently nanosuspensions and gold nanoparticles phyto-formulated using NSS extract have demonstrated significant antioxidant and antidiabetic activity (292, 293). In addition, an array of nanotechnological carriers have come to the fore which could potentially overcome the poor solubility and bioavailability of orally administered TQ (280, 294), delivering a high payload of TQ via the oral route by mixing it with relatively non-toxic solvents like DMSO is also an option, and not just limited to diabetes treatment (295). Recently, synthetic analogs of TQ have come to the fore with greater efficacy and safety than the natural version, but these have been chiefly used against cancer and other diseases and not for diabetes treatment (284, 296, 297). TQ’s chemical and biological transformation has yielded derivatives with enhanced antioxidant potential (298, 299). Given the limited supply of natural TQ (NS being the primary source), escalating future needs may have to be supplied through synthetic versions. The performance of synthetic TQ analogs, which surpass the natural version in oncology experiments, is very encouraging, but whether this holds true for diabetes remains to be seen (75).

### Aspects of TQs mode of action

**Figure 3:**
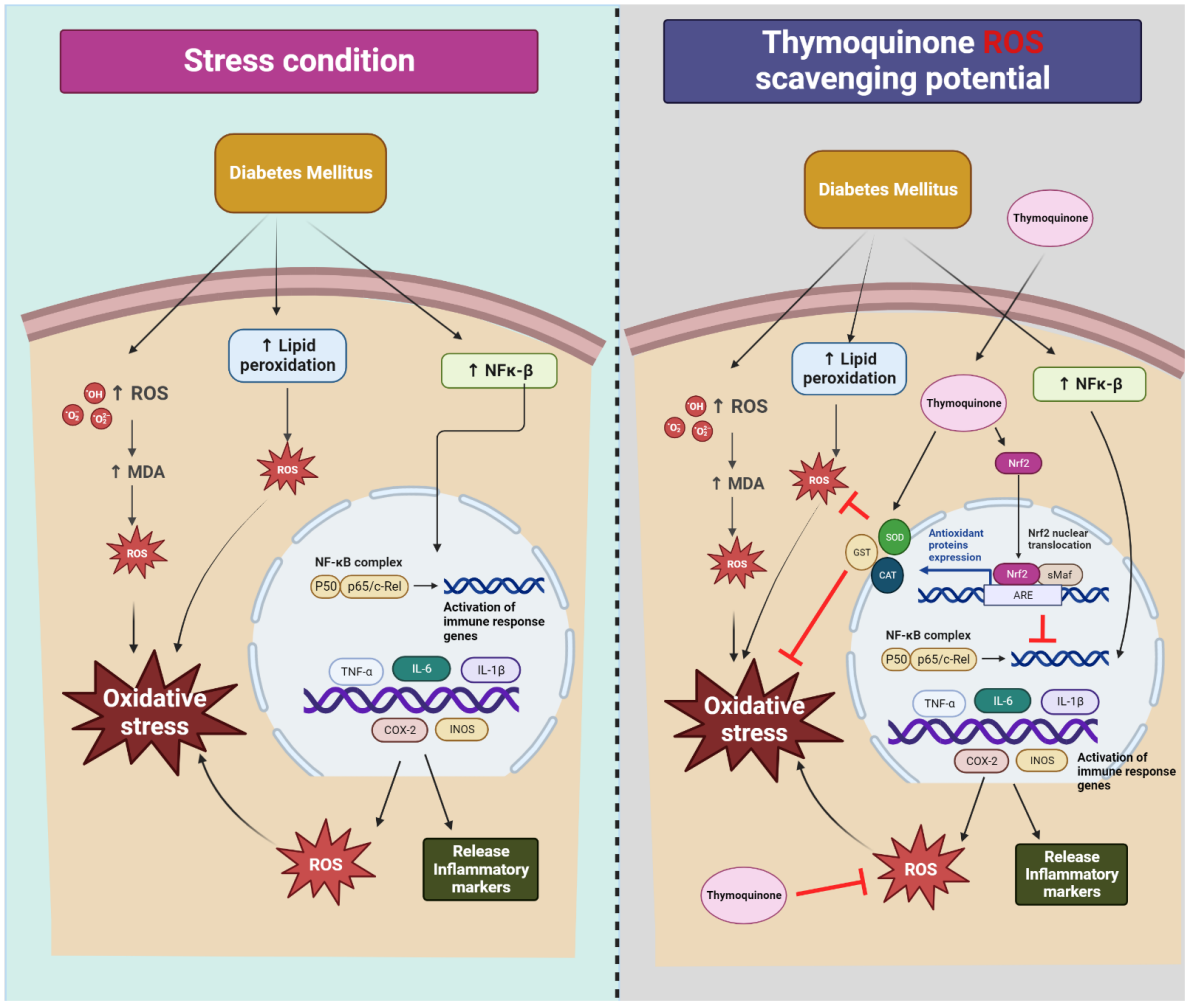
Mechanistic aspects of oxidative stress and its mitigation by thymoquinone.

Although the high levels of TQ used in animal research have raised some safety concerns, its usage in mixtures by people for more than a millennium has been relatively incident-free (100) (Table 2). TQ’s non-toxicity and safety makes it ideal for consideration as a pharmacological agent with substantial therapeutic and commercial potential (296). Increased oxidant levels and lipid peroxidation are hallmarks of diabetes (300), and TQ can directly scavenge ROS such as superoxide (O_2_^−^), hydroxyl radicals (OH^−^) and hydrogen peroxide (H_2_O_2_) that cause OS (87) (Figure 3). Its ability to quench free radicals matches that of SOD (179, 301), although it is not so effective against hydroxyl and 2,2′-diphenyl-p-picrylhydrazyl (DPPH) radicals (302, 305). TQ has been shown to suppress lipid peroxidation, reduce intracellular MDA (302, 303), and enhance antioxidant defenses by non-enzymatically augmenting the activity of antioxidant enzymes (304). Several studies have demonstrated that TQ increased the level and activity of both the primary antioxidant enzymes, SOD, CAT, and glutathione S-transferase (GST), and the secondary antioxidant enzymes like glutathione reductase (GR) and GPx (303, 305, 306). Its ability to react *in vivo* with such antioxidant enzymes, especially GSH, and form more potent quenching moieties that replenish and eventually replace the endogenous antioxidant system is critical in fighting OS-mediated pathogenesis (285). TQ is also able to check the auto-oxidation of glucose to prevent the runaway generation of NFκ-B-mediated ROS and proinflammatory cytokines typical of DM onset (307). Because of TQ’s propensity to react with thiol-rich proteins and modulate powerful antioxidant enzymes like GSH, it is bundled with an exclusive group of therapeutic drugs called Michael reaction acceptors (308) that are associated with safeguarding overall cellular health (239, 309) and is a natural activator of Nrf2 signaling. In the presence of OS, Nrf2 induced many of the cell’s antioxidant enzymes while simultaneously repressing NF-κB, IL-1β, IL-6, TNK-z, COX-2, iNOS, TGF-β1, and NOX4, which decreased inflammation, DNA and mitochondrial damage (310). The possibility of using TQ to augment the new diabetic therapy based on the gut hormone incretin has gained much currency because of its few side effects compared to conventional drugs and the absence of any other herbal derivatives that demonstrably modulate incretin (GLP-1) (248, 311, 312).

NS produces ample amounts of TQ, but problems with the preparation of NS extract, non-standardization of testing parameters, disease severity, and duration of NS dosing could account for the conflicting claims where clinical trials have failed to find a role for NS in lowering MDA levels. Variable TQ content in commercially available NS products is another confusing issue in their varied efficacy, with requests for regulating them for TQ content (313, 314). A new study suggests that a TQ content of 30 mg per day be tested in a therapeutic context (314). Significant diversity in NSO chemotypes is also a concern, as those from Turkey and Egypt are classified as TQ phenotypes with the highest TQ content, whereas others, such as those from the subcontinent, are more mixed (p-cymene/TQ), and some may have phenylpropanoids instead of TQ, the so-called trans-anethole chemotype (315, 316). Similarly, care needs to be exercised when interpreting NS’s *in vivo* antioxidant efficacy based on TAC measurements since it is an *in vitro* assay that measures only non-enzymatic antioxidant capacity, whereas NS has both (107, 253, 317).

### Genetic impact of NS supplementation in DM

Genotype and environment both play a role in the etiology of T2DM (318, 319), and there has been strong interest in establishing the genetic triggers of OS in diabetes (276).

#### Insulin-like growth factor 1

NS has potent antidiabetic activity, as reflected by the up-regulation of several essential genes, such as insulin-like growth factor-1 and IGF-1. IGF-1 is widely present in mammalian tissues, and its functions include regulating metabolism and enhancing tissue development and growth (320). IGF-1 manifests its effects by binding specific receptors on target cells, stimulating glucose uptake, lowering blood glucose, and improving insulin sensitivity (321). Its mechanism of action appears independent of insulin receptor activation, but NSS has been shown to induce hypoglycemia by upregulating the IGF-1 gene and reducing DM-induced OS (322). IGF-1 improves insulin resistance in T2DM and in patients with more severe insulin resistance, where clinical trials have demonstrated the potential utility of IGF-1 in ameliorating clinical symptoms (157, 323, 324).

In diabetes, loss of insulin responsiveness can occur because some elements of insulin signaling pathways, such as insulin receptors, are disrupted. NSO upregulates insulin-signaling pathways and augments the expression of insulin-like growth factor-1 (IGF-1), inducing the signaling molecule, protein kinase B (Akt), and activating glucose transporter-4 (GLUT4). GLUT4 is then translocated to the plasma membrane, where it imports glucose into the cell (325). Dysfunctional GLUT-4 has been linked to insulin resistance (326). Thus, NS can decrease insulin resistance by improving tissue sensitivity to insulin action (327, 328), presumably in concert with its suppression of insulin clearance via inhibition of insulin-degrading enzymes (IDEs). Greater insulin sensitivity is also linked to NSO’s ability to lower triglyceride levels (329).

#### DM-associated endothelial dysfunction

Endothelial dysfunction is described as an “impairment of the ability of the endothelium to maintain vascular homeostasis” properly and is the principal underlying reason for DM-associated vascular pathologies (330). DM is associated with reduced expression and activity of endothelial nitric oxide synthase (eNOS), which is central to maintaining cardiovascular tone and function (149, 331). TQ improves endothelial function by inhibiting OS and stabilizing the renin-angiotensin (RAS) system (332). The proliferation and migration of vascular smooth muscle cells (VSMCs) is a characteristic of endothelial dysfunction in diabetes, and inhibitory drugs are highly sought after. Animal studies show TQ’s antiproliferative and anti-migratory effect on VSMCs through the AMPK/ Peroxisome proliferator-activated receptor gamma PPARγ/ Peroxisome proliferator-activated receptor-gamma coactivator (PGC-1α) pathway (333). TQ reduces vascular inflammation by repressing the expression of vascular endothelial growth factor (VEGF) and monocyte chemo-attractant protein-1 (MCP-1), besides lowering levels of cytokines IL-6 and IL-8 in human vascular endothelial cells (HUVECs) (334). TQ has been shown to reverse endothelial dysfunction by increasing NO generation and bioavailability. Vascular cell adhesion protein-1 (VCAM-1) is involved in the adhesion of lymphocytes, eosinophils, and basophils to the vascular endothelium and is an essential mediator in developing atherosclerosis and DM. VCAM-1 can recruit monocytes to the sites of atherosclerotic lesions, initiating and developing vascular inflammation (335, 336). *Vcam-1* gene expression was upregulated in the aortic tissue of diabetic rats, while NS seeds significantly reduced *vcam-1* gene expression in the aorta, potentially reducing vascular inflammation and restoring endothelial function (149). TQ interfered with TNF-α signaling to modulate IL-6 and IL-8 expression and downregulated IL-8 and ICAM-1/VCAM-1 expression in rheumatoid arthritis (337). TQ also downregulated toll-like receptor-2 (TLR-2) and-4, which are crucial to the vascular inflammation of diabetic microangiopathy (338, 339), underscoring its potential for controlling and managing DM.

#### Effects on lectin-like oxidized low-density lipoprotein receptor-1

The LOX-1 LDL receptor is an essential element in the progression of atherosclerosis through its intimate relationship with CV dysfunction and DM pathogenesis. Experiments with diabetic rats showed upregulation of LOX-1 expression in the vascular endothelium of the aorta. LDL uptake by the LOX-1 receptor triggers many pathophysiological changes, such as decreased eNOS activity and stimulation of adhesion molecule expression (340, 341). NSS extract inhibited *lox-1* gene expression in aortic tissue (342). LDL binding to the LOX-1 receptor increased OS, decreased NO production, potentiated superoxide generation, and activated NF-kB-all of which exacerbate DM pathogenesis. Additional studies on human aortic endothelial cells demonstrated that high glucose levels increased *lox-1* gene expression; thus, down-regulating LOX-1 using NSS extract could potentially suppress the pathophysiological processes related to LOX-1 (343, 344), restore normal endothelial function and decrease vascular complications in DM (149).

#### NS and TQ suppression of cyclooxygenase-2

COX-2 continues to be a target for repression because of its central role in inducing the inflammatory processes associated with diabetes and metabolic syndrome (345, 346). The classical non-steroidal inflammatory drugs (NSAIDs) such as salicylates and the newer, more selective COXIBs (an NSAID sub-category of COX-2 inhibitors) have been used, but their side effects have initiated a search for safer, natural COX inhibitors (221). Culinary spices, such as NS, have garnered much attention because of their anti-inflammatory potential (347). Among the NS-derived benzoquinones, both TQ and its partially reduced form, hydroquinone, stand out as potent inhibitors of COX-2, while thymol selectively inhibited COX-1 (221). TQ demonstrated efficacy against autoimmune diseases, including T1DM, by repressing COX, LOX, cytokines, lipid peroxidation, and IL-1. ROS activates the Nuclear factor kappa-light-chain-enhancer of activated B cells (NF-κB) (348), increasing DM-related inflammation (349, 350). Many inflammatory response factors such as adhesion molecules, inflammatory enzymes, proinflammatory cytokines, and chemokines are products of genes regulated by NF-κB (351, 352), including the inflammatory COX-2. COX-2 is a multiplex enzyme with cyclooxygenase and peroxidase activities that generate ROS and cause OS (353, 354). NS and TQ treatment of streptozotocin (STZ)-induced diabetic rats significantly suppressed the COX-2 expression in pancreatic tissue. The treatments that decreased COX-2 mRNA also reduced pancreatic tissue lipid peroxidation and MDA levels and increased SOD activity (355).

### The synergy of NS and gut microbiota

The link between gut microbiota and the onset and incidence of type 1 and type 2 DM has been unequivocally established, triggered by the leaky gut condition arising from altered gut microbiota (356, 357). The microbial population in the gut is complex, unique to the individual, and plays a vital role in the body’s physiological and metabolic processes. Extensive studies of the gut bacterial community have revealed species predominantly belonging to the phyla, Firmicutes, Bacteroidetes, Actinobacteria, Proteobacteria, and Verrucomicrobia (10, 357). Gut bacteria are vulnerable to diet and stress (357), and a dysbiotic population contributes to the onset of T2DM through an altered synthesis of short-chain fatty acids, gut hormone levels, bile acids, and branched-chain amino acid metabolism (358). Metf, the mainstream glucose-lowering medication for DM, ameliorates gut dysbiosis (359, 360). Many treatments exist for diabetes, but its complexity has made curing it a challenge (202, 357), emphasizing the need for testing novel therapeutic approaches (358). Modulating the gut microbiota through pre-and pro-biotics for the complementary treatment and management of T2DM has recently gained much currency (361, 362). Several recent reviews have focused on the potential of next-generation probiotic candidates as adjuncts to conventional probiotics such as lactobacilli and bifidobacteria to offset the harmful consequences of DM (363). The antioxidant activity of a probiotic *Lactobacillus* species was identified as the reason for its hypoglycemic effect (364). Combining probiotics with existing allopathic antidiabetic medications and plant-based nutraceuticals is an exciting research avenue (357, 365). Co-administering NS with probiotics has yielded fruitful results in treating unrelated human disorders (366). One proposed mechanism of the antibacterial effect of NS and TQ against a variety of Gram-positive and Gram-negative bacteria is by targeting them with ROS while at the same time protecting host tissues from OS through antioxidants (284) and selectively allowing probiotic LAB species to flourish (367). This activity holds promise for T2DM patients once their gut becomes populated with opportunistic pathogens such as *Clostridium* spp., *E. coli,* and betaproteobacteria (368). Reversing diabetes-related gut dysbiosis through plant extracts such as those from NSS is of great scientific interest (202). High doses of NSS polysaccharides have been used to promote Bacteroidetes over Firmicutes to alleviate T2DM (202), and the use of beneficial yeast strains like *Saccharomyces boulardii* is also promising (369).

## Conclusion

This review (1985 to January 2022) discusses the latest findings on diabetic pathogenesis and its treatment, emphasizing the herb *Nigella sativa* and its active constituent, thymoquinone. An obvious outcome of this narrative is that the different experimental designs used by various investigators mean that conclusions about its interventional potential should be interpreted with care. Despite many publications, the medically relevant dosage of TQ and NS supplements remains contentious. The multi-modal nature of NS fractions and TQ is minutely examined for their antidiabetic effects and verified in the lab, and clinical trials make their therapeutic potential as a complementary medication patently clear. However, the pharmacokinetic interaction of NS with conventional drugs raises some concerns which need addressing. Nevertheless, the review clarifies the need to assess NS and its products in clinical trials using diabetic patients with reasonable glycemic control but vascular complications.

## Author contributions

AS: co-developed the concept, data collection, analysis, and preliminary draft writing. AZ: co-developed the concept, data collection and analysis, and improved and finalized the manuscript. HA: validated the data analysis. NK: did part of the data collection and analysis. All authors contributed to the article and approved the submitted version.

## Figures and Tables

**Table:** 

NS	*Nigella sativa*
DM	Diabetes mellitus
TQ	Thymoquinone
ID	Insulin-dependent
NID	Non-insulin dependent
T2DM	Type 2 diabetes mellitus
BPA	Bisphenol A
WHO	World Health Organization
USFDA	United States Food and Drug Administration’s
CAM	Complementary and alternative medicine
ROS	Reactive oxygen species
Metf	Metformin
GLP-1	Glucagon-like peptide-1
TAIM	Traditional Arab and Islamic medicine
GRAS	Generally regarded as safe
NSS	NS seeds
NSO	NS oil
T1DM	Type 1 diabetes mellitus
MAPKs	Mitogen-activated protein kinase
AMPK	Adenosine monophosphate-activated protein kinase
SLGT1	Sodium-glucose linked transporter 1
SOD	Superoxide dismutase
CAT	Catalase
GPx	Glutathione peroxidase
LDL;	Low density lipoprotein
HDL-C	High density lipoprotein cholesterol
AST	Aspartate aminotransferase
ALT	Alanine transaminase
ALP	Alanine phosphate
APAP	Acetaminophen
GSSG	Oxidized glutathione
MDA	Malondialdehyde
GSH	Reduced glutathione
RNS	Reactive nitrogen species
NADPH	Nicotinamide adenine dinucleotide phosphate
OH	Hydroxyl radical
O-2	Superoxide anion
H_2_O_2_	Hydrogen peroxide
NO	Nitric oxide
TAC	Total antioxidant capacity
AGEs	Advanced glycation end product
RAGE	Receptor for AGE
DT	Dihydro-thymoquinone
GLUT-4	Glucose transport-4
COX	Cyclooxygenase
LOX	Lipoxygenase
IL-1β	Interleukin-1β
TNF-α	Tumor necrosis factor-alpha
INF-γ	Interferon-gamma
IL-6	Interleukin-6
MAGE	Melibiose-derived AGE
HbA1c	Glycated hemoglobin
DMSO	Dimethyl sulfoxide
DPPH	2,2′-Diphenyl-p-pierylhydrazyl
GST	Glutathione S-transferase
GR	Glutathione reductase
IGF-1	Insulin-like growth factor
eNOS	Endothelial nitric oxide synthase
RAS	Renin-angiotensin
VSMC	Vascular smooth cells
VEGF	Vascular endothelial growth factor
MCP-1	Monocyte chemo-attractants protein-1
HVECs	Human vascular epithelial cells
VCAM-1	Vascular cell adhesion protein-1
LOX-1	Lectin-like oxidized low-density lipoprotein receptor-1
CV, NSAIDS	Non-steroidal inflammatory drugs
NF-κB	Nuclear factor-kappa B
STZ	Streptozotocin
Akt	Protein kinase B
IDSs	Insulin degrading enzymes
LAB	Lactic acid bacteria
